# Two Different Phospholipases C, Isc1 and Pgc1, Cooperate To Regulate Mitochondrial Function

**DOI:** 10.1128/spectrum.02489-22

**Published:** 2022-11-15

**Authors:** Maria Balazova, Petra Vesela, Lenka Babelova, Ivana Durisova, Paulina Kanovicova, Jakub Zahumensky, Jan Malinsky

**Affiliations:** a Department of Membrane Biochemistry, Institute of Animal Biochemistry and Genetics, Centre of Biosciences, Slovak Academy of Sciences, Bratislava, Slovakia; b Department of Functional Organization of Biomembranes, Institute of Experimental Medicine, Academy of Sciences of the Czech Republic, Prague, Czech Republic; Université Côte d’Azur, CNRS, Inserm

**Keywords:** phospholipase C, phosphatidylglycerol, phosphatidylethanolamine, diacylglycerol, ceramide, respiration

## Abstract

The absence of Isc1, the yeast homologue of mammalian neutral sphingomyelinase type 2, leads to severe mitochondrial dysfunction. We show that the deletion of another type C phospholipase, the phosphatidylglycerol (PG)-specific phospholipase Pgc1, rescues this defect. Phosphatidylethanolamine (PE) levels and cytochrome *c* oxidase activity, which were reduced in *isc1*Δ cells, were restored to wild-type levels in the *pgc1*Δ *isc1*Δ mutant. The Pgc1 substrate PG inhibited the *in vitro* activities of Isc1 and the phosphatidylserine decarboxylase Psd1, an enzyme crucial for PE biosynthesis. We also identify a mechanism by which the balance between the current demand for PG and its consumption is controlled. We document that the product of PG hydrolysis, diacylglycerol, competes with the substrate of PG-phosphate synthase, Pgs1, and thereby inhibits the biosynthesis of excess PG. This feedback loop does not work in the absence of Pgc1, which catalyzes PG degradation. Finally, Pgc1 activity is partially inhibited by products of Isc1-mediated hydrolysis. The described functional interconnection of the two phospholipases contributes significantly to lipid homeostasis throughout the cellular architecture.

**IMPORTANCE** In eukaryotic cells, mitochondria are constantly adapting to changes in the biological activity of the cell, i.e., changes in nutrient availability and environmental stresses. We propose a model in which this adaptation is mediated by lipids. Specifically, we show that mitochondrial phospholipids regulate the biosynthesis of cellular sphingolipids and vice versa. To do this, lipids move by free diffusion, which does not require energy and works under any condition. This model represents a simple way for the cell to coordinate mitochondrial structure and performance with the actual needs of overall cellular metabolism. Its simplicity makes it a universally applicable principle of cellular regulation.

## INTRODUCTION

Together with glycerophospholipids and sterols, sphingolipids represent one of the main classes of lipids forming the biological membranes of all eukaryotic organisms. Besides their essential structural function, sphingolipids and their precursors, long-chain bases and ceramides, participate in cell signaling during polarized growth, cell cycle control, and adaptation to various types of environmental and endoplasmic reticulum (ER) stresses, etc. (see reference 1 and references therein; 2, 3). In higher eukaryotes, including humans, dysregulated sphingolipids and especially ceramides underlie severe pathologies, including type 2 diabetes, cardiovascular diseases, and cancer (4–6). In cellular metabolism, ceramides are generated either during sphingolipid neosynthesis or by the hydrolysis of complex sphingolipids.

In yeast, complex sphingolipids (inositol phosphoceramides and their mannosylated variants) are hydrolyzed to ceramides by a single inositol phosphosphingolipid phospholipase C, Isc1, an enzyme homologous to mammalian neutral sphingomyelinase type 2 (7, 8). During fermentation, Isc1 localizes to the membrane of the endoplasmic reticulum. During the diauxic shift, the protein changes its localization toward the outer mitochondrial membrane (9). The translocation of Isc1 to mitochondria and increases in its activity are induced following protein phosphorylation by Sch9 kinase (10).

Sch9 itself requires dual phosphorylation to activate its kinase function, combining signals from the membrane stress-detecting eisosome complex at the plasma membrane (11, 12) and nutrient-responding TORC1 at the membrane of the vacuole (13, 14). The eisosomal branch of this signaling pathway includes Pkh1/2 kinases and the eisosome core protein Pil1 (15, 16). Sch9 activity stabilizes the ceramide pool through the combined action of sphingolipid degradation and neosynthesis pathways. Specifically, it not only stimulates the Isc1-mediated hydrolysis of complex sphingolipids but also represses the expression of genes encoding ceramidases, *YDC1* and *YPC1* (10). The importance of this balanced regulation is well illustrated by the data obtained from *isc1*Δ cells, in which both TORC1- and Pkh1-mediated phosphorylations of Sch9 contribute to their respiration defects (see references 17 and 18, respectively).

Ceramides generated by Isc1 contribute to normal mitochondrial function. The increased Isc1 activity in cells after the diauxic shift positively correlates with increased levels of the product of Isc1-catalyzed complex sphingolipid hydrolysis, phytoceramide, in these cells. At the same time, both effects are conditioned by running phosphatidylglycerol (PG) synthesis in mitochondria (19). PG is a tightly regulated, low-abundance phospholipid in yeast, predominantly serving as a precursor in the cardiolipin (CL) biosynthetic pathway. The Pgs1-catalyzed synthesis of PG-phosphate (PGP), which takes place at the inner mitochondrial membrane, represents a rate-limiting step of the pathway. Following biosynthesis, PG is either utilized as a substrate by the CL synthase Crd1 or spread to other cellular membranes, where it is subjected to rapid degradation by a specific phospholipase C, Pgc1 (20).

The supply of exogenous phytoceramide was enough to rescue the growth defect of cells lacking either Isc1 or the PGP synthase Pgs1 (19). However, at least in the latter strain, this could not be related to the rescue of the mitochondrial function because PG and CL, anionic phospholipids indispensable for respiration (21), were still absent. In any case, the excess ceramide was somehow able to participate in improving the phenotype of not only *isc1*Δ but also *pgs1*Δ cells. As mentioned above, in wild-type diauxic cells, the increased ceramide content depends on PG synthesis (19). In this study, we asked whether elevated PG levels themselves could at least partially rescue the phenotype of *isc1*Δ cells. Deletion of the *PGC1* gene has been shown previously to induce PG accumulation in various backgrounds (20, 22, 23). Therefore, we constructed a *pgc1*Δ *isc1*Δ double-deletion mutant. A detailed characterization of this double-deletion strain reveals a tight interconnection between the mechanisms of sphingolipid and phospholipid metabolism regulation.

## RESULTS AND DISCUSSION

### Respiration defects of *isc1*Δ cells can be partially rescued by PGC1 deletion.

The *pgc1*Δ *isc1*Δ double-deletion mutant was constructed as described in Materials and Methods. First, we analyzed the mitochondrial phospholipid content in the newly constructed *pgc1*Δ *isc1*Δ strain to test whether it accumulated PG as expected. To do this, we compared the phospholipid compositions of mitochondrial membranes of the wild-type, *isc1*Δ, *pgc1*Δ, and *pgc1*Δ *isc1*Δ strains. As mentioned above, the transition of Isc1 from the ER to mitochondria is controlled by Sch9 (10). To distinguish the effect of the complete absence of Isc1 from the effect of the lack of a change in its local distribution, we also included the *sch9*Δ mutant in this comparison. To further refine the distinction between the effect of the absence of the Pgc1 protein itself and the loss of its function and, thus, the effect of PG accumulation, we also included PG-accumulating mutants defective in CL synthesis, *crd1*Δ, *crd1*Δ *isc1*Δ, and *crd1*Δ *pgc1*Δ.

**FIG 1:**
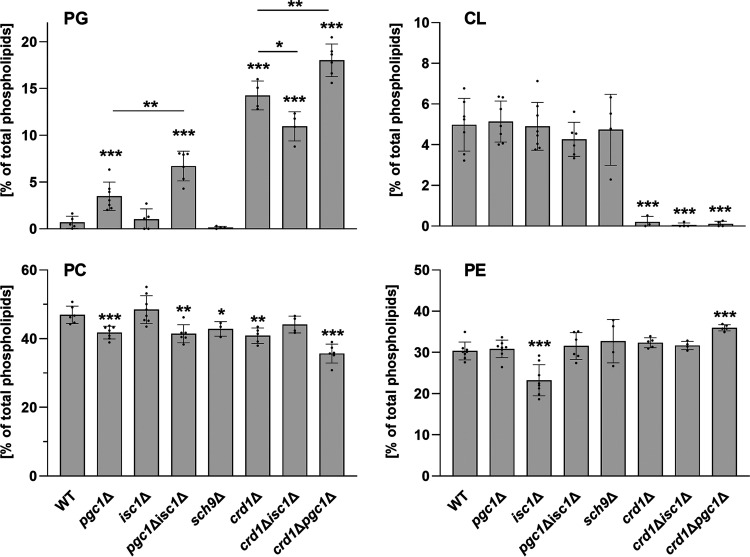
Phospholipid content analysis. The indicated strains of S. cerevisiae (see Table S1 in the supplemental material for details) were cultivated in SMDGE medium for 24 h. Lipids from the mitochondrial fractions were extracted and separated, and the relative amounts of phospholipids were calculated based on the contents of inorganic phosphate. Data represent mean values from at least 4 independent experiments (dots) ± SD (error bars). Statistically significant differences between mutant strains and the wild type (WT), between the *pgc1*Δ *isc1*Δ and *pgc1*Δ strains, between the *crd1*Δ *isc1*Δ and *crd1*Δ strains, or between the *crd1*Δ *pgc1*Δ and *crd1*Δ strains are indicated by asterisks (*, *P* < 0.05; **, *P* < 0.01; ***, *P* < 0.001). CL, cardiolipin; PC, phosphatidylcholine; PE, phosphatidylethanolamine; PG, phosphatidylglycerol.

The results of the phospholipid content analysis are shown in Fig. 1. As expected, we detected a remarkable accumulation of PG in *pgc1*Δ *isc1*Δ cells. In fact, this mutant accumulated about twice as much PG as the *pgc1*Δ strain did. We did not identify any significant difference in cardiolipin contents among the analyzed strains. However, we found significantly decreased phosphatidylethanolamine (PE) contents in the mitochondria of the *isc1*Δ strain. This drop in PE levels was not observed in *sch9*Δ cells, leading us to conclude that this was not due to the loss of Isc1 translocation into mitochondria, as neither of these mutants contained the mitochondrial fraction of the protein, but rather was due to a general lack of Isc1 protein function in *isc1*Δ cells. In addition, wild-type-like PE levels could be restored by *PGC1* deletion in the *isc1*Δ strain. In accordance with previously reported data, we also found lower phosphatidylcholine (PC) contents in the two strains lacking the *PGC1* allele (23), and a small but statistically significant decrease in PC levels was also detected in the *sch9*Δ strain. We hypothesize that the restoration of PE levels in the *pgc1*Δ *isc1*Δ mutant may be due to a reduction in PC synthesis, as PE serves as a substrate here. The lower demand for the substrate could lead to a relative increase in its levels. Similar to the *PGC1* deletion, mutants lacking the *CRD1* gene also accumulate PG. However, there was no statistically significant difference in the amounts of PE and PC between *crd1*Δ *isc1*Δ mutant and wild-type cells (Fig. 1).

**FIG 2:**
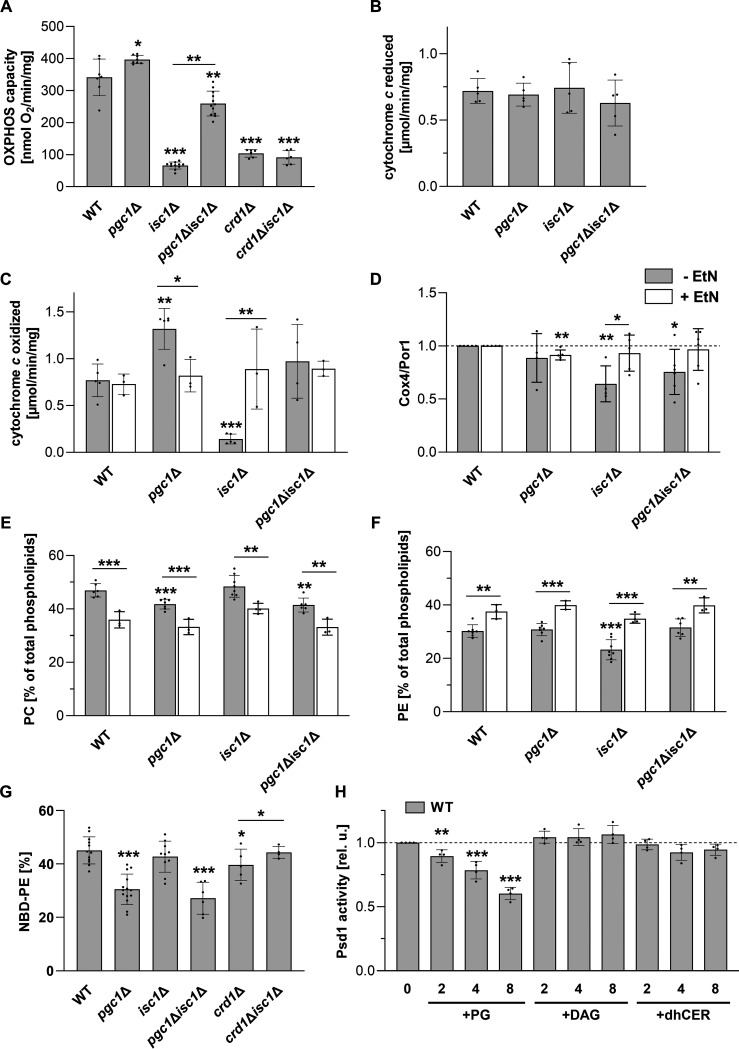
Role of Pgc1 and Psd1 in the reduced respiration of *isc1*Δ cells. The indicated strains of S. cerevisiae (see Table S1 in the supplemental material for details) were cultivated in SMDGE medium for 24 h without (gray bars) (at least 4 independent experiments) or with (white bars) (3 independent experiments) added ethanolamine (EtN) (final concentration, 2 mM). (A) Mitochondria isolated from these cells were used to measure oxygen consumption in the presence of ADP (OXPHOS capacity), with NADH as a respiratory substrate. (B and C) The *in vitro* activities of complex III (B) and complex IV (C) were measured in lysates prepared from isolated mitochondria (see Materials and Methods for details). (D) The relative levels of Cox4 (subunit of complex IV) were normalized to the Por1 level. (E and F) Lipids from the mitochondrial fractions were extracted and separated, and the relative amounts of PC (E) and PE (F) were calculated based on the contents of inorganic phosphate. (G) The *in vitro* phosphatidylserine decarboxylase activity of Psd1 was measured in mitochondrial fractions using NBD-PS as a substrate. The fluorescent product (NBD-PE) was quantified. (H) The dependence of Psd1 activity on the concentration of phosphatidylglycerol (PG), diacylglycerol (DAG), or dihydroceramide (dhCER) (see Materials and Methods for details) in the reaction mixture (numbers denote the concentrations of added lipids in micrograms per reaction mixture) was measured in mitochondrial fractions of wild-type (WT) cells. Relative activities are presented: values detected in samples with no added lipids (41.7% ± 2.2% NBD-PE) were normalized to 1. Data represent mean values from at least 4 independent experiments (dots) ± SD (error bars). Statistically significant differences between mutant strains and the wild type or between samples with and without added ethanolamine are indicated in panels A to G, and statistically significant differences compared to the sample with no added lipids are indicated in panel H (*, *P* < 0.05; **, *P* < 0.01; ***, *P* < 0.001). rel. u., relative units.

Next, we compared the mitochondrial functions in the analyzed strains. Specifically, we measured the respiratory capacities of mitochondria isolated from wild-type, *isc1*Δ, *pgc1*Δ, and *pgc1*Δ *isc1*Δ cells in the ADP-activated state in the presence of NADH (oxidative phosphorylation [OXPHOS] capacity). Consistent with previously reported data, we found that the OXPHOS capacity was reduced to ~20% of the wild-type value in the *isc1*Δ strain (17, 19, 24) and was significantly increased in the *pgc1*Δ strain (22, 23). In the *pgc1*Δ *isc1*Δ double mutant, the OXPHOS capacity was partially restored to 76% ± 11% of the wild-type value. Only a weak restoration effect was observed for the *crd1*Δ *isc1*Δ mutant (27% ± 6% of the wild-type value) (Fig. 2A). Cytochrome *c* reductase and cytochrome *c* oxidase (complexes III and IV, respectively) activities measured *in vitro* revealed that the observed changes in the OXPHOS capacity are due mainly to changes in complex IV activity. While no significant variations in complex III activity were detected among the strains analyzed, complex IV activity decreased to 18% ± 7% in *isc1*Δ mitochondria and was nearly doubled in *pgc1*Δ mitochondria. In the double mutant, complex IV activity was restored to the level of the wild-type strain (Fig. 2B and C). These differences in complex IV activity only partially correlated with the observed differences in the amounts of the complex IV subunit Cox4 among the strains analyzed. In particular, the recovery of complex IV activity in the *pgc1*Δ *isc1*Δ double mutant cannot be explained by differences in protein abundance alone (Fig. 2D).

PE is a nonbilayer lipid that, besides CL, is important for mitochondrial structure and function (25, 26). The defect in *de novo* PE synthesis can be bypassed by the supply of exogenous ethanolamine during cultivation (27). We therefore tested whether the ethanolamine supply can affect the *isc1*Δ phenotype. A comparison of the phospholipid profiles showed that for all strains analyzed, cells grown in medium supplemented with ethanolamine contained a higher PE fraction at the expense of the PC fraction (Fig. 2E and F). In addition, the complex IV activity in *isc1*Δ cells fed with ethanolamine was restored to wild-type levels (Fig. 2C). We conclude that the deficiency in complex IV in *isc1*Δ cells is due to a reduced PE fraction in their mitochondrial membranes. A similar observation that a moderate reduction in PE levels of <30% induces severe alterations in mitochondrial morphology and the function of complex IV was reported previously in mice (28).

Both defects found in the *isc1*Δ mutant, i.e., the decreased PE content and the drop in complex IV activity, are also generated in cells lacking the mitochondrial phosphatidylserine (PS) decarboxylase Psd1 (29), mediating the crucial step of the major pathway of PE synthesis (30). To determine whether the phenotype of *isc1*Δ cells is due to impaired Psd1 function, we compared the mitochondrial PS decarboxylase activities of the analyzed strains (Fig. 2G). No significant decrease in Psd1 activity could be detected in the mitochondrial fraction isolated from *isc1*Δ cells, which indicated that PS decarboxylase deficiency is not the primary reason for the *isc1*Δ phenotype. In contrast, we found significantly decreased Psd1 activity in mitochondrial fractions of both strains lacking *PGC1* (Fig. 2G), which did not affect the PE contents in these cells (Fig. 1), and normal or even increased activity of complex IV was detected in the mitochondrial fractions of *pgc1*Δ *isc1*Δ and *pgc1*Δ cells, respectively (Fig. 2C). The modulation of Psd1 function thus does not appear to be the cause of the rescue of the *isc1*Δ phenotype by *PGC1* deletion (Fig. 1 and Fig. 2A to C).

We searched for the reason why the strains lacking the *PGC1* gene exhibited decreased PS decarboxylase activity. A similar, although less pronounced, effect was observed with the *crd1*Δ strain. Both *PGC1* and *CRD1* deletions lead to massive PG accumulation (22, 31) (Fig. 1). We therefore asked whether the increased PG content could be sufficient to reduce the rate of PS decarboxylation. We measured the *in vitro* PS decarboxylase activity in isolated wild-type mitochondria (Psd1 activity) supplemented with different amounts of PG. As documented in Fig. 2H, the Psd1 activity gradually decreased with increasing amounts of PG in the reaction mixture. We conclude that the reduced PS decarboxylase activity documented in Fig. 2G was a consequence of PG accumulation in Pgc1-deficient cells.

### Diacylglycerol inhibits PG synthesis.

**FIG 3:**
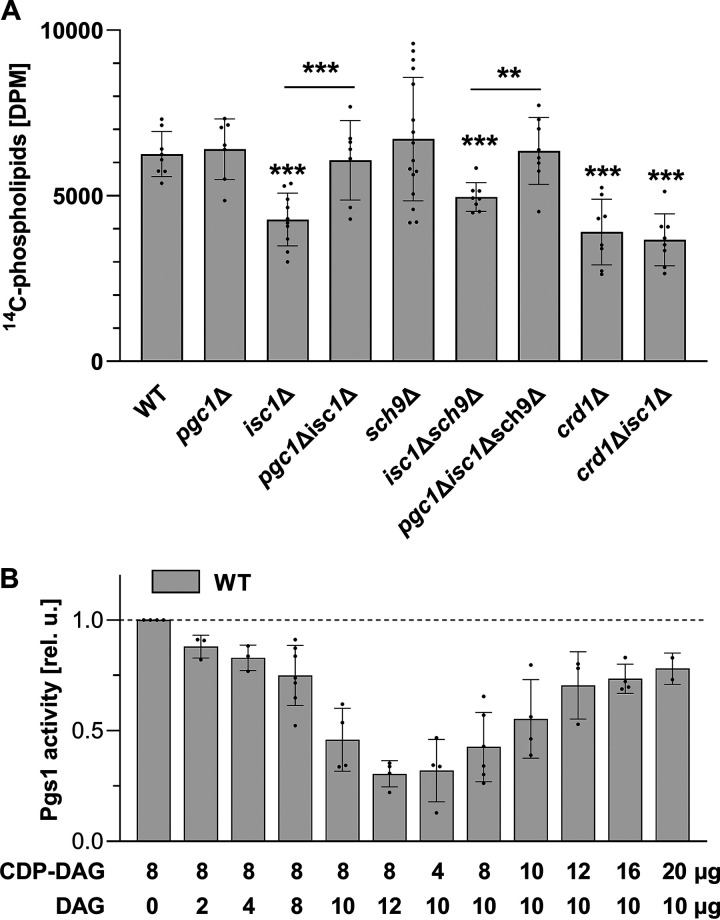
Regulation of Pgs1 activity. The indicated strains of S. cerevisiae (see Table S1 in the supplemental material for details) were cultivated in SMDGE medium for 24 h. (A) The *in vitro* Pgs1 activity in mitochondrial fractions was measured by the amount of radioactive ^14^C incorporated into phospholipids, using ^14^C-labeled glycerol-3-phosphate as a substrate (see Materials and Methods for details). (B) The dependence of Pgs1 activity on the concentration of CDP-diacylglycerol (DAG), DAG, or their combinations in the reaction mixture was measured in the mitochondrial fractions of wild-type (WT) cells. Relative activities are presented: values detected in samples with no added lipids (6,212 ± 889 DPM) were normalized to 1. (numbers denote the concentrations of added lipids in micrograms per reaction mixture). Data in panels A and B represent mean values from at least 3 independent experiments (dots) ± SD (error bars). Statistically significant differences between mutant strains and the wild type, between the *pgc1*Δ *isc1*Δ and *isc1*Δ strains, or between the *pgc1*Δ *isc1*Δ *sch9*Δ and *isc1*Δ *sch9*Δ are indicated by asterisks (*, *P* < 0.05; **, *P* < 0.01; ***, *P* < 0.001). DPM, disintegration per minute.

The accumulation of PG under normal conditions is efficiently prevented by Pgc1 activity (31). The immediate intensity of Pgc1-mediated PG degradation can be temporarily enhanced by mobilizing the phospholipase pools from storage sites on lipid droplets (20). However, a prolonged coexistence of excess PG synthesis and its intense degradation would be energetically demanding for the cell. We therefore asked whether there is some form of feedback regulation that would prevent excessive PG synthesis in cases where PG degradation is already active. As mentioned above, PG synthesis is rate limited by the activity of Pgs1. Could it be that Pgs1 somehow senses ongoing Pgc1 activity? In an *in vitro* assay, we detected no difference in Pgs1 activities between the wild-type and *pgc1*Δ strains. However, the significantly reduced Pgs1 activity in *isc1*Δ cells was restored to the wild-type levels by *PGC1* deletion (Fig. 3A). Of note, the reduction in Pgs1 activity caused by the absence of *ISC1* was independent of the presence of Sch9, as Pgs1 activity comparable to that of the wild type was detected in the *sch9*Δ strain. Similarly, the restoration of Pgs1 activity following *PGC1* deletion occurred in both the *isc1*Δ and *isc1*Δ *sch9*Δ backgrounds, i.e., again independently of Sch9. This suggests that Pgs1 activity in *isc1*Δ cells is reduced due to a loss of Isc1 function in general and not due to a change in the cellular localization of Isc1, which is thought to be driven by Sch9 (10). Finally, we detected low Pgs1 activity in the *crd1*Δ strain, and the *CRD1* deletion was not able to restore the reduced Pgs1 activity of the *isc1*Δ strain (Fig. 3A).

The mechanism of the proposed ability of Pgc1 to modulate Pgs1 activity was investigated in more detail. The molecular function of Pgc1 is to hydrolyze PG to diacylglycerol (DAG) and glycerol-3-phosphate (31). Pgs1 transfers the phosphatidyl group from CDP-DAG to the hydroxyl group of glycerol-3-phosphate (32) to synthesize PGP, which is rapidly dephosphorylated to PG. In our *in vitro* analysis, we detected maximal Pgs1 activity in the presence of 8 μg of the CDP-DAG substrate (Fig. 3B). This activity gradually decreased upon the addition of increasing concentrations of DAG to the reaction mixture. This negative correlation occurred specifically for DAG and did not occur upon the addition of other lipids or upon increasing the amount of the substrate (CDP-DAG) alone. No lipid other than the substrate contributed to the Pgs1 activity. Negligible Pgs1 activity was detected in the absence of CDP-DAG (see Fig. S1A in the supplemental material). Finally, the DAG-inhibited Pgs1 activity was recovered by the addition of larger amounts of CDP-DAG, indicating competition between the two compounds (Fig. 3B).

In this context, it is worth mentioning that the recovery of Pgs1 activity by the *PGC1* deletion is not a new phenomenon. We reported recently that the reduced Pgs1 activity in a strain lacking the monolyso-CL transacylase Taz1 was fully recovered in a *pgc1*Δ *taz1*Δ double-deletion mutant. Analogously to the *pgc1*Δ *isc1*Δ strain, the *pgc1*Δ *taz1*Δ strain accumulated PG. Consistent with our recently reported data, it could be that the lower level of DAG production associated with the *PGC1* deletion led to the observed restoration of Pgs1 activity in *pgc1*Δ *taz1*Δ cells (23).

Taken together, our data suggest that the Pgc1-mediated PG hydrolysis product DAG is enough to reduce Pgs1 activity by competitively inhibiting CDP-DAG binding to the enzyme. DAG freely diffuses across biological membranes (33, 34) and could thus represent an effective tool for a cell to mediate both the signalization of increased PG degradation, i.e., reduced PG demand, and the self-regulation of PG production by Pgs1.

### Functional interplay of phospholipases Pgc1 and Isc1.

**FIG 4:**
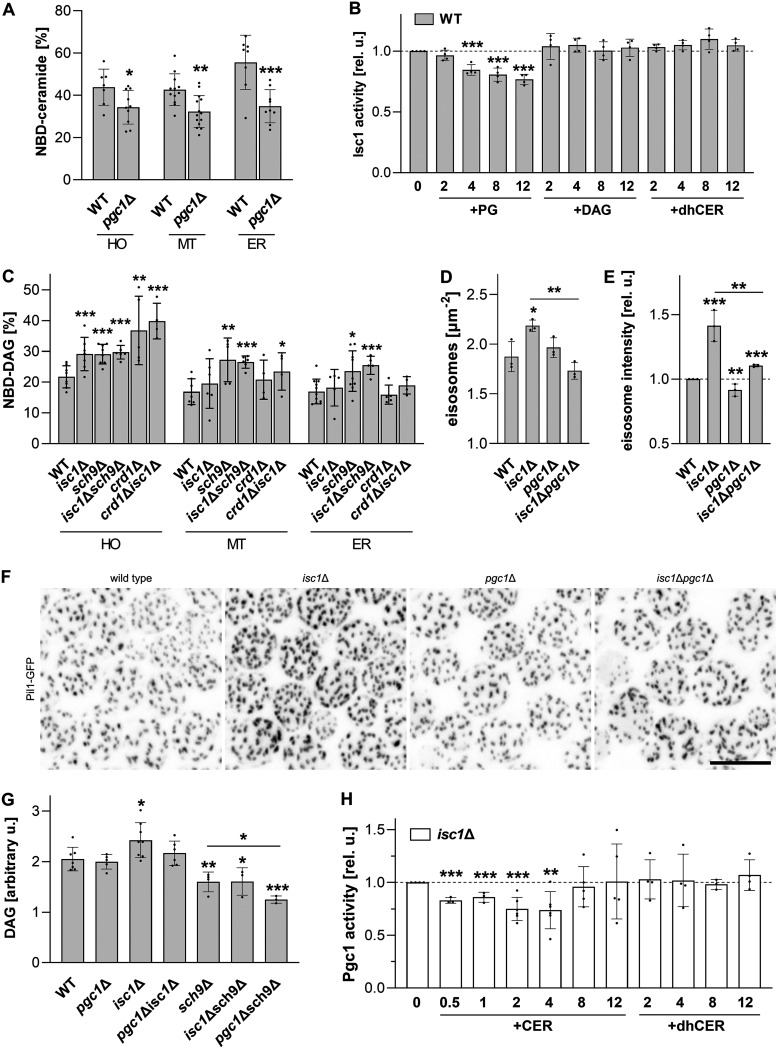
Regulation of Isc1 and Pgc1 activities. The indicated strains of S. cerevisiae (see Table S1 in the supplemental material for details) were cultivated in SMDGE medium for 24 h. (A to C and H) The *in vitro* activities of Isc1 (A and B) and Pgc1 (C and H) were measured in the whole-cell homogenate (HO), isolated mitochondria (MT), and the ER fraction (A and C) or after the addition of the indicated concentrations (in micrograms per reaction mixture) of selected lipids to the mitochondrial fractions. Relative activities are presented in panels B and H: values detected in samples with no added lipids (39.9% ± 12.1% NBD-ceramide [CER] and 36.4% ± 5.0% NBD-diacylglycerol [DAG], respectively) were normalized to 1. (D to F) The structures of the plasma membrane-associated eisosomes in the wild-type (WT), *isc1*Δ, *pgc1*Δ, and *pgc1*Δ *isc1*Δ strains expressing Pil1-GFP were compared (see Materials and Methods for details). The eisosome density and fluorescence intensity were analyzed (D and E) in maximum projections of six consecutive confocal sections (F). Bar, 5 μm. At least 250 cells/strain were analyzed in each experiment. (G) Lipids from the mitochondrial fractions were extracted and separated, and the amounts of DAG were compared. Data represent mean values from at least 4 independent experiments (dots) ± SD (error bars). Statistically significant differences between mutant strains and the wild type, between the *pgc1*Δ *isc1*Δ and *isc1*Δ strains, or between the *pgc1*Δ *sch9*Δ and *isc1*Δ strains are indicated in panels A, C to E, and G, and statistically significant differences compared to the sample with no added lipid are indicated in panels B and H (*, *P* < 0.05; **, *P* < 0.01; ***, *P* < 0.001). PG, phosphatidylglycerol; dhCER, dihydroceramide.

It has been documented that Isc1 is activated *in vitro* by the anionic phospholipids PG, CL, and PS (35). We asked whether the excess PG that accumulated in cells lacking the *PGC1* gene is sufficient to increase the Isc1 activity in the *pgc1*Δ strain. Measurements of Isc1 activity *in vitro* showed that under conditions of respiratory growth, the opposite is true, and Isc1 activity is significantly decreased in the whole-cell homogenate as well as the ER and mitochondrial membrane fractions isolated from *pgc1*Δ cells, compared to the wild type (Fig. 4A). To verify that the reduction in Isc1 activity observed in the *pgc1*Δ strain was indeed due to excess PG, we compared the Isc1 activities in mitochondria isolated from these cells grown in medium with and without inositol. Inositol is a known repressor of PG synthesis (36, 37), and cells with the *PGC1* gene deletion grown in inositol-supplemented medium contain amounts of PG similar to those of the wild type (31). Consistent with this, we detected wild-type Isc1 activity in mitochondria isolated from *pgc1*Δ cells grown in inositol-containing medium. Of note, the effect of PG-suppressed Isc1 activity was independent of the presence of Sch9 (Fig. S1B).

A similar effect of reduced Isc1 activity could be observed when excess PG was added to the membrane samples isolated from wild-type cells. No change in Isc1 activity was observed upon the addition of other lipids (Fig. 4B). The relative decrease in the Isc1 activity observed at higher concentrations of PG was comparable to the difference between the enzyme activities in mitochondria isolated from wild-type and *pgc1*Δ cells (compare Fig. 4A and B). We conclude that Pgc1 participates in Isc1 activation by preventing PG accumulation. Similarly, the increase in Isc1 activity observed during the diauxic shift (19) can be interpreted as a release from PG inhibition under conditions of an increased need for PG as a major precursor in CL biosynthesis at the onset of respiration.

The observed inhibitory effect of PG on Isc1 activity is in clear contrast to previous reports of Isc1 activation *in vitro* by anionic phospholipids, including PG (35). We believe that this is primarily because previous studies used glass beads to disrupt cells, which may have compromised the integrity of the inner membranes, which were further challenged by the use of detergents and external anionic phospholipids (PS) in all of their assays of Isc1 activity (7, 19, 35). Our conclusion that PG inhibits Isc1 function could be supported by the existence of a common C-terminal motif in the Isc1 and Psd1 protein sequences that binds PS and possibly also PG and cardiolipin (35). Such domain homology suggests a common mechanism of regulation of Psd1 and Isc1 by PG, and we have shown an inhibitory effect of PG on Psd1 activity (Fig. 2G and H). The activity of Pgc1 itself is also tightly regulated. A large pool of inactive Pgc1 is stored in lipid droplets and is activated only after release to the inner cellular membranes (20), where it is also rapidly degraded by the endoplasmic reticulum-associated protein degradation (ERAD) pathway (38). The molecular mechanism for the timely release of Pgc1 from storage to the places of its enzymatic activity remains to be identified. However, since this step involves the translocation of the enzyme from the lipid monolayer on the surface of the lipid droplet to the lipid bilayers of various subcellular membranes (20), it is reasonable to expect that it is directly influenced by the lipid composition of the target membranes. For example, the Isc1-catalyzed hydrolysis of complex sphingolipids reduces these tightly exoplasmically localized lipids to ceramide, which diffuses between membrane leaflets (39). Thus, Isc1 activity can substantially alter the properties of the ER and/or outer mitochondrial membranes in which active Pgc1 has been localized (20). We therefore asked whether Isc1 could play a role in the regulation of Pgc1 function.

First, we compared the Pgc1 activities in the wild-type and *isc1*Δ strains. We detected increased Pgc1 activity in the whole-cell homogenate of *isc1*Δ cells. This increase was not detected in either the mitochondrial or ER fraction (Fig. 4C), which suggested that it could reflect a changed Pgc1 activity in other cellular compartments of the *isc1*Δ mutant. In the genome-wide screen, *isc1*Δ cells exhibited hyperassembled eisosomes at their plasma membrane (11). We checked whether this particular phenotype of the *isc1*Δ mutant could also be rescued by the deletion of *PGC1*. To this end, we compared the eisosomal patterns of wild-type, *isc1*Δ, *pgc1*Δ, and *pgc1*Δ *isc1*Δ cells, which were visualized using the fluorescently labeled eisosome organizer Pil1. Quantitative analysis revealed statistically significant increases in the eisosome density and Pil1-green fluorescent protein (GFP) fluorescence intensity in the *isc1*Δ mutant. For both parameters, this increase could be compensated for by the deletion of *PGC1* (Fig. 4D to F).

Consistent with the increased Pgc1 activity, we also detected increased levels of the Pgc1-catalyzed hydrolysis product DAG in *isc1*Δ cells. DAG levels returned to the wild-type value in the *pgc1*Δ *isc1*Δ strain, indicating that Pgc1 activity was indeed responsible for this effect (Fig. 4G). Moreover, the increase in DAG correlated markedly with the decreased Pgs1 activity in the *isc1*Δ strain (Fig. 3A). We consider this as further evidence that physiological lipid levels (ceramides and DAG) are able to regulate the activity of the enzymes involved (Pgc1 and Pgs1, respectively). Contrary to our expectation, there was no statistically significant decrease in the DAG content in the *pgc1*Δ strain compared to the wild-type value. However, the situation was different in the absence of the Sch9 protein. In *sch9*Δ cells, sphingolipid profiling showed reduced levels of many ceramide species (10). It was therefore not surprising that similar to *isc1*Δ cells lacking the ceramide-generating lipase, we detected increased Pgc1 activity in the *sch9*Δ strain. In contrast to the *isc1*Δ strain, this increase was statistically significant in all cell fractions analyzed (Fig. 4C). The total DAG content in *sch9*Δ mitochondria was lower than that in the wild type, and it could not be elevated in this strain even by the deletion of the *ISC1* gene. However, it was decreased further in the absence of Pgc1, suggesting that, in contrast to the wild type and similar to the *isc1*Δ strain, a considerable fraction of DAG in *sch9*Δ cells is the result of the Pgc1-mediated hydrolysis of PG. This difference between the wild type on one side and the *isc1*Δ and *sch9*Δ backgrounds on the other side illustrates the versatility and adaptability of lipid-mediated enzyme regulation. While ceramide production by Isc1 was critical for Pgc1 activity in the wild type, in the absence of the protein in the *isc1*Δ strain or in the environment with deregulated sphingolipid biosynthesis in the *sch9*Δ strain, ceramide levels were out of the range significantly regulating Pgc1 activity, and the contribution of Isc1 in the latter case was not essential. Conversely, the regulatory potential of Pgc1-generated DAG was dominant in both mutant strains, where it effectively reduced Pgs1 activity, and became ineffective in the wild-type strain (Fig. 3A).

Next, we asked whether Pgc1 activity in isolates from *isc1*Δ cells could be attenuated to wild-type levels by adding exogenous ceramide to the *in vitro* assay mixture. Indeed, a significant decrease in Pgc1 activity was detected when small amounts of ceramide were added to the reaction mixture, with the lowest Pgc1 activity being detected after the addition of 2 μg of ceramide (Fig. 4H). Further increases in the amounts of ceramide in the reaction mixture led to a gradual disappearance of its inhibitory effect. The increasing variance among biological replicates of the high-ceramide samples suggested that the addition of large amounts of ceramide affected the structure of the membrane isolates. One possible explanation could be lipid-phase separation. This effect was highly specific as no significant change in Pgc1 activity was observed after the addition of C_16_ dihydroceramide (dhCER) (Fig. 4H) or other lipid species (Fig. S1C). The differential effects of distinct ceramide species on the phenotype of *isc1*Δ cells were described previously (e.g., see reference 19), but further experiments will be required to explain the molecular details of the observed phenomenon.

**FIG 5:**
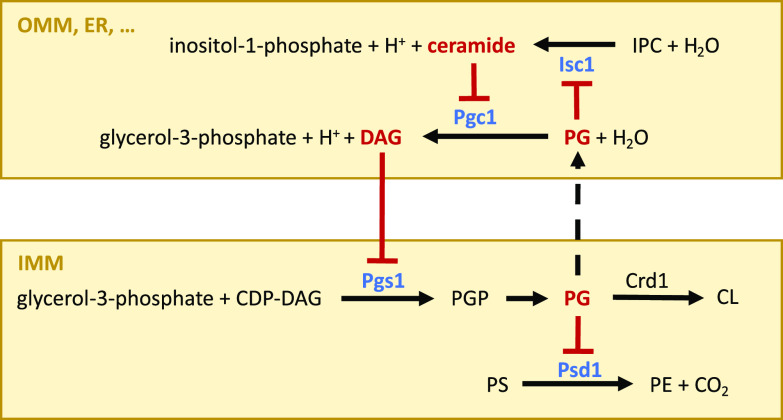
Mechanism for the mutual regulation of Isc1 and Pgc1 phospholipases. Our data indicate that two lipids, DAG and PG, inhibit the key enzymes of mitochondrial phospholipid biosynthesis: (i) the PGP synthase Pgs1, (ii) the phosphatidylserine decarboxylase Psd1, and (iii) the inositol phosphosphingolipid phospholipase C Isc1. The latter is of particular importance because ceramide, a product of Isc1-catalyzed hydrolysis, inhibits the PG-specific phospholipase C Pgc1, which degrades PG to form DAG. The entire loop ensures the efficient control of PG (CL) and PE synthesis in the context of environmental stress stimuli affecting the sphingolipid biosynthetic pathway. OMM, outer mitochondrial membrane; IMM, inner mitochondrial membrane; IPC, inositol phosphoceramide.

Two different type C phospholipases, Isc1 and Pgc1, share subcellular localizations at the ER and the outer mitochondrial membrane (9, 20). In this study, we have shown that there is also a functional connection between them because the activity of one influences the activity of the other and vice versa. In this feedback loop, the ceramide generated by Isc1 activity suppresses the degradation (Fig. 4C) and stimulates the synthesis (Fig. 3A) of the CL precursor PG by inhibiting the enzymatic activities of Pgc1 and, subsequently, Pgs1. Feedback is provided by the PG itself because higher levels of this particular lipid inhibit Isc1 activity (Fig. 5). This direct link between mitochondrial lipid metabolism and the sphingolipid biosynthesis pathway, which responds to a wide range of environmental cues (40, 41), could be of high physiological relevance.

In addition to the regulation of CL biosynthesis, actual PG levels may also affect the production of PE (Fig. 2G and H), another nonbilayer lipid that plays a key role in mitochondrial morphology and function and whose biological relevance largely overlaps that of CL (26, 42). This type of PE regulation could be important in CL deficiency. We have previously shown that despite the accumulation of large amounts of PG in the inner mitochondrial membrane of the *crd1*Δ strain, Pgc1 maintains low PG levels in other cellular membranes (20). Consistent with this, we measured increased Pgc1 activity in mitochondria of the *crd1*Δ and *crd1*Δ *isc1*Δ strains (although in the *crd1*Δ strain, the detected increase was below the level of statistical significance) (Fig. 4C). The regulatory mechanism proposed here (Fig. 5) predicts that the increased Pgc1 activity results in the increased production of DAG, which could contribute to Pgs1 inhibition in strains lacking the *CRD1* gene (Fig. 3A). The fact that the observed increase in Pgc1 activity was more pronounced in the *crd1*Δ *isc1*Δ strain than in the *crd1*Δ strain correlates with the lower degree of PG accumulation in the double mutant (Fig. 1) and the partial inhibition of Psd1 activity observed in the *crd1*Δ strain but not the *crd1*Δ *isc1*Δ strain (Fig. 2G). It has been suggested that in the absence of CL, PG fulfills its function in binding respiratory complexes (e.g., see reference 43). Thus, it is likely that this bound PG cannot inhibit Psd1, which could account for the increased PE levels. This is also why the inhibitory effect of the accumulated PG on the Psd1 activity is low in the *crd1*Δ mutant, compared to the effect on the *pgc1*Δ strain, which has normal CL levels.

The proposed model for the lipid-mediated regulation of mitochondrial phospholipid homeostasis (Fig. 5) represents a simple way for the cell to ensure optimal mitochondrial structure and function in response to current needs. The fact that its functionality is based purely on the spontaneous movement of lipids along a concentration gradient, whether by lateral diffusion or by intermembrane exchange, means, among other things, that the coupling of individual regulatory steps does not require energy and works under any condition, including nutrient deprivation and stress, etc. This makes it a universally applicable principle of cellular regulation.

## MATERIALS AND METHODS

### Yeast strains and growth conditions.

All Saccharomyces cerevisiae strains used in this study are listed in Table S1 in the supplemental material. Cell cultures were grown in complex medium (yeast extract-peptone dextrose [YPD] [2% peptone, 1% yeast extract, 2% glucose]). For experiments, cells were grown aerobically at 30°C for 24 h to the diauxic shift in a defined synthetic medium prepared as previously described (SMDGE, synthetic medium containing 0.2% D-glucose, 3% glycerol, and 1% ethanol as a carbon source) (43). If not stated otherwise, SMDGE medium lacked inositol.

### Strain construction.

*pgc1*Δ::*HIS3* mutant strains of both mating types were prepared by the replacement of the *pgc1*Δ::*KanMX4* disruption cassette with *pgc1*Δ::*HIS3*, the *crd1*Δ::*HIS3* mutant strain was prepared by the replacement of the *crd1*Δ::*KanMX4* disruption cassette with *crd1*Δ::*HIS3*, and the *sch9*Δ::*NatMX4* mutant strain was prepared by the replacement of the *sch9*Δ::*KanMX4* disruption cassette with *sch9*Δ::*NatMX4* in the *pgc1*Δ, alpha-*pgc1*Δ, and *sch9*Δ collection strains, respectively, as described previously (44). The *pgc1*Δ *isc1*Δ, *isc1*Δ *sch9*Δ, and *crd1*Δ *isc1*Δ double mutants were prepared by the transformation of *pgc1*Δ::*HIS3*, *sch9*Δ::*NatMX4*, and *crd1*Δ::*HIS3*, respectively, with a disruption cassette, *isc1*Δ::*KanMX4*, PCR amplified from the *isc1*Δ strain. Yeast transformation was performed by the lithium acetate method (45). The *pgc1*Δ *isc1*Δ *sch9*Δ triple mutant was selected by tetrad analysis after the crossing of two parental strains, alpha-*pgc1*Δ::*HIS3* and *isc1*Δ *sch9*Δ, as isogenic to BY4741, except for the three required deletions. To visualize Pil1-GFP *in vivo*, the wild-type, *isc1*Δ, *pgc1*Δ, and *pgc1*Δ *isc1*Δ strains were transformed with the YIp128-*PIL1-GFP* plasmid. The plasmid was constructed by inserting the *PIL1* gene as a HindIII-BamHI fragment into the YIp128-GFP integrative plasmid. Prior to transformation, the plasmid was linearized by cleavage with XbaI to enable its genome integration. Successful transformants were selected based on the loss of leucine auxotrophy due to the *PIL1*::*GFP*::*LEU2* insert.

### Phospholipid extraction and analysis.

Lipids were extracted from the mitochondrial fractions of the analyzed cells at a concentration corresponding to 0.8 mg in 3.5 mL of chloroform-methanol-HCl (60:30:0.26, vol/vol/vol) for 1 h with shaking. Residual contaminants were removed by additional washes of the organic phase with 3.5 mL of a 0.1 mM MgCl_2_ (wt/vol) solution. Individual phospholipids were separated by thin-layer chromatography (TLC) on silica gel 60 plates (Merck) using a chloroform-methanol-acetic acid developing solvent (65:25:8, vol/vol/vol). Phospholipids were visualized on thin-layer chromatography plates by staining with iodine vapor. After the evaporation of the iodine, a thin-layer chromatography plate was moistened with ultrapure water, and each phospholipid spot was scraped off, put into a glass tube, and dried in an oven at 105°C. After cooling, 200 μL of H_2_SO_4_-HClO_4_ (9:1, vol/vol) was added, and the samples were incubated at 200°C for 30 min. The samples were cooled down at room temperature and then incubated with 4.8 mL of a solution containing 0.26% (wt/vol) (NH_4_)_6_Mo_7_O_24_. 4H_2_O–ANSA (4-amino-3-hydroxy-1-naphthalenesulfonic acid, in the following mixture: 16% [wt/vol] K_2_S_2_O_5_, 0.252% [wt/vol] C_10_H_9_NO_4_S, 0.5% [wt/vol] Na_2_SO_3_) at a ratio of 250:11 (vol/vol) at 105°C for a further 30 min. After incubation, the samples were cooled and vortexed, and the silica gel was gently spun down at 500 × *g* for 2 min. The inorganic phosphate (P_i_) of phospholipids reacts with ammonium heptamolybdate, forming molybdenum blue, the concentration of which was assessed by absorption spectrometry at 830 nm.

### Enzymatic assays.

Intact mitochondria were isolated from cells grown to the diauxic shift as described previously (22). The final mitochondrial pellet was suspended in respiration buffer (0.6 M mannitol, 20 mM HEPES-KOH [pH 7.1], 2 mM MgCl_2_, 1 mM EGTA, 0.1% fatty acid-free bovine serum albumin, 10 mM KH_2_PO_4_) and used for measurements of O_2_ consumption (OXPHOS capacity) as described previously (23); the activities of cytochrome *c* reductase and cytochrome *c* oxidase were measured as described previously (22).

The activity of Pgc1 was measured as described previously (20), with modifications in the amounts of isolated subcellular fractions used. Briefly, a mixture containing 40 μL of 0.3 M Tris-HCl (pH 7.4), 20 μL of NBD-PG (12-[(7-nitro-2-1,3-benzoxadiazol-4-yl)amino]dodecanoyl-PG, 0.8 μg), and the respective subcellular fractions (cell homogenate, mitochondrial fraction, or ER fraction at a concentration corresponding to 50 μg of proteins) was incubated at 30°C for 40 min. The linearity of the reaction under these conditions was verified previously (20). The reaction was stopped by the addition of chloroform-methanol-HCl (2 mL; 100:100:0.6, vol/vol/vol) and 1 mL of water to the mixture. After separation, the organic phase was dried under a stream of nitrogen. NBD-labeled lipids were separated by one-dimensional thin-layer chromatography for 15 min using a chloroform-methanol developing solvent (70:35, vol/vol). The degradation of the exogenous fluorescent substrate NBD-PG to NBD-DAG was quantified using a TLC scanner (460 nm; Camag), and the phospholipase C activity of Pgc1 was determined as a fraction of NBD-DAG in the total NBD-labeled lipids.

The activity of Pgs1 was determined by the quantification of the incorporation of the radiolabeled substrate [^14^C]glycerol-3-phosphate into chloroform-soluble products, as described previously (23). Briefly, a reaction mixture containing 50 mM morpholineethanesulfonic acid (MES)-HCl (pH 7.0), 0.1 mM MnCl_2_, synthetic lipids at the indicated concentrations (if not stated otherwise, 0.083 mM CDP-DAG was used, which corresponded to 8 μg of the lipid per reaction mixture), 1 mM Triton X-100, 0.02 mM [^14^C]glycerol-3-phosphate (40,000 cpm/nmol), and the mitochondrial fraction corresponding to 25 μg of mitochondrial protein in a total volume of 120 μL was incubated for 20 min at 30°C. The reaction was stopped by the addition of excess chloroform-methanol-HCl (100:100:0.6) to the mixture, and phase separation was then induced by water. Aliquots of the organic layer were evaporated under a stream of nitrogen and dissolved in a scintillation mixture, and the radioactivity of each sample was determined with a scintillation counter.

Isc1 activity was determined by the quantification of the fluorescently labeled product NBD-ceramide after the incubation of different cell fractions with the fluorescently labeled substrate NBD-sphingomyelin. Briefly, fresh cell fractions (cell homogenate, 20 μg of proteins; mitochondrial fraction, 15 μg of proteins; microsomal fraction enriched by the ER, 10 μg of proteins) were incubated in 100 μL of buffer containing 100 mM Tris-HCl (pH 7.4), 5 mM MgCl_2_, and 0.5 μg of NBD-sphingomyelin. The mixture was incubated at 30°C for 20 min. The reaction was stopped by the addition of chloroform-methanol-HCl (2 mL; 100:100:0.6, vol/vol/vol) and 1 mL of water to the mixture. After separation, the organic phase was dried under a stream of nitrogen. NBD-labeled lipids were separated by one-dimensional thin-layer chromatography for 15 min using a chloroform-methanol developing solvent (70:35, vol/vol). The separated NBD-labeled lipids were scanned with a TLC scanner (Camag) in the fluorescence mode at a wavelength of 460 nm, and the phospholipase activity of Isc1 was determined as a fraction of NBD-ceramide, a product of NBD-sphingomyelin hydrolysis, in the total NBD-labeled lipids.

The activity of Psd1 was determined by the quantification of the fluorescently labeled product NBD-PE after the incubation of the mitochondrial fraction with NBD-PS as the substrate. The 140-μL reaction mix contained 100 mM Tris-HCl (pH 7.4), 10 mM EDTA, 0.8 μg NBD-PS, and the cell homogenate (40 μg of proteins) or the mitochondrial fraction (15 μg of proteins). After a 20-min incubation at 30°C, the reaction was stopped, and the mixture was separated and quantified in the same way as described above for Isc1 activity. The overall decarboxylase activity was determined as a fraction of NBD-PE in the total NBD-labeled lipids.

### Synthetic lipids.

In the *in vitro* assays testing the dependence of the activities of various enzymes on specific lipids, the following synthetic lipid molecules (Merck) were added to the reaction mixture: 1-palmitoyl-2-oleoyl-*sn*-glycerol (DAG) (catalogue number 800815), *N*-octanoyl-d-*erythro*-sphingosine (CER) (catalogue number 860645), *N*-palmitoyl-d-*erythro*-sphinganine (dhCER) (catalogue number 860634), 1-palmitoyl-2-oleoyl-*sn*-glycero-3-phospho-(1′-*rac*-glycerol) (PG) (catalogue number 810218), and 1,3-dihexadecanoyl-2-(*cis*-9-octadecenoyl)glycerol (TAG) (catalogue number D2157). Lipids were solubilized in chloroform-methanol (2:1, vol/vol). Before use, the required amount of lipids in the solution was added to a Pyrex tube and dried under a stream of nitrogen. The dried lipids were resuspended in the appropriate buffer described above.

### Fluorescence microscopy.

Yeast cells in a culture grown in SMDGE medium for 24 h at 30°C were concentrated by brief centrifugation, immobilized on a 0.17-mm cover glass by a thin film of 1% agarose prepared in 50 mM potassium phosphate buffer (pH 6.3), and observed using an LSM 880 confocal laser scanning microscope (Zeiss) equipped with a 100× PlanApochromat oil immersion objective (numerical aperture [NA], 1.4). The fluorescence signal of GFP (excited by the 488-nm line of an Ar laser) was detected using a photomultiplier tube after filtering with a 493- to 550-nm-band-pass emission filter. Three-dimensional (3D) stacks of confocal sections were acquired with sampling in the axial direction of 0.7 μm. Maximum-intensity projections were calculated and quantitative analysis of confocal images was performed with ImageJ 1.53c software (U.S. National Institutes of Health, MD, USA) using custom-made macros developed for this study (available at https://github.com/jakubzahumensky/Isc1_paper [the repository also contains sample images]).

### DAG analysis.

Lipids from the mitochondrial fraction prepared by Zymolyase treatment (corresponding to 1 mg of proteins) were extracted, dried under a nitrogen stream, resuspended in a chloroform-methanol mixture (2:1), and separated by TLC as described previously (46). Following sulfuric acid staining, the relative lipid content was determined using Camag WinCATS software after scanning TLC plates on Camag TLC scanner 3 at 475 nm.

### Miscellaneous.

The preparation of the microsomal fraction enriched by the ER was performed as described previously (20). Western blot analysis of the abundance of Cox4 was performed as described previously (22). Briefly, isolated mitochondria were lysed, separated on a 12% denaturing polyacrylamide gel, and blotted onto a nitrocellulose membrane. The membrane was blocked with 5% milk in Tris-buffered saline (TBS) buffer (50 mM Tris-HCl [pH 8.0], 150 mM NaCl, 0.05% [vol/vol] Tween 20) overnight and immunostained using primary rabbit anti-Cox4 (1:1,000) or anti-Por1 (1:5,000) antibody for mitochondrial porin and secondary anti-rabbit antibody (Sigma). Secondary antibody was visualized using an ECL+ kit (Amersham). Statistical comparisons were carried out by one-way analysis of variance using SigmaPlot 14 software (Systat Software, San Jose, CA). All graphs (GraphPad Prism 9 software; GraphPad Software, San Diego, CA) show the means ± standard deviations (SD).
