# Supplementary material for: Two Different Phospholipases C, Isc1 and Pgc1, Cooperate To Regulate Mitochondrial Function
Source: Microbiol Spectr. 2022 Nov 15;10(6):e02489-22. doi: 10.1128/spectrum.02489-22 (PMC9769635; doi:10.1128/spectrum.02489-22)
Supplement: Supplemental file 1 — Supplemental material. Download spectrum.02489-22-s0001.pdf, PDF file, 0.7 MB [file spectrum.02489-22-s0001.pdf]

Supplemental Material for Spectrum02489-22

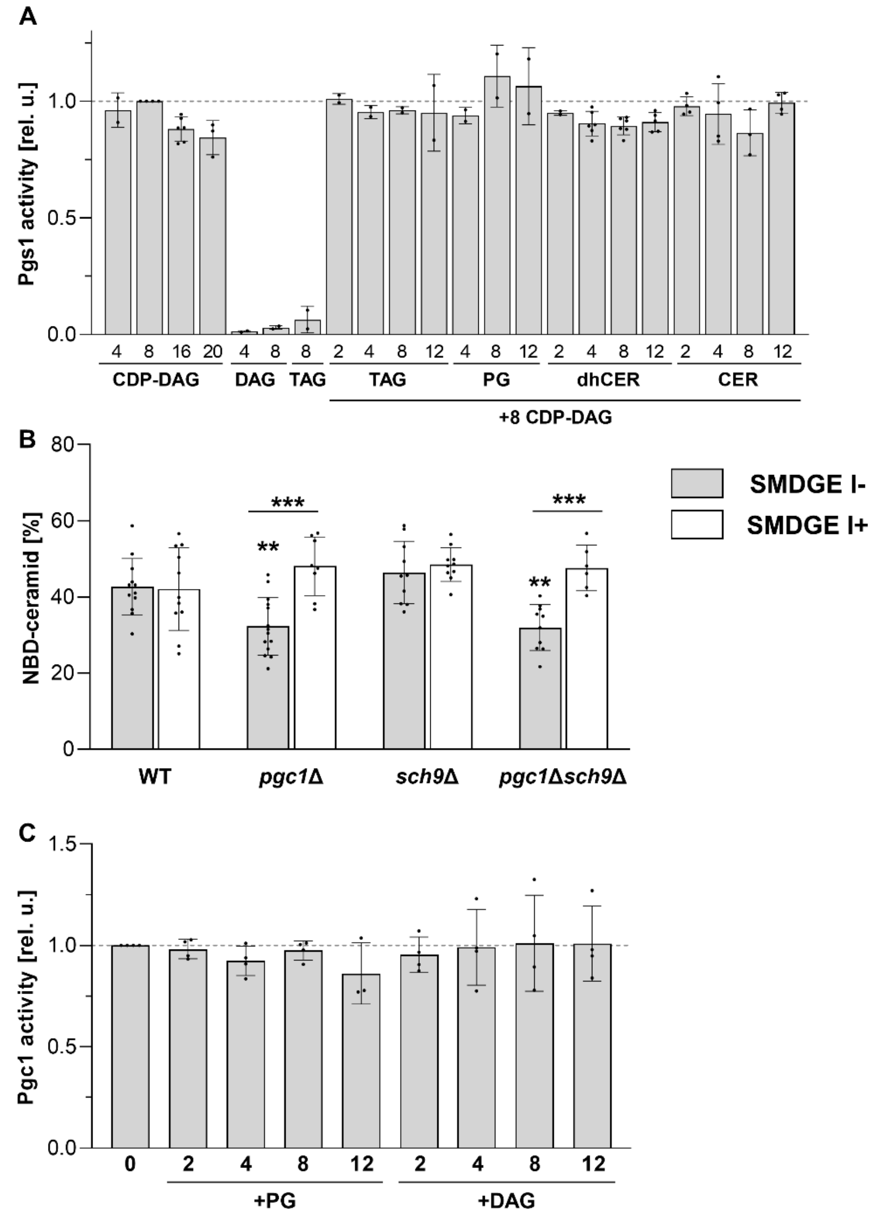

Figure S1 Further indications for regulation of Pgs1, Isc1 and Pgc1 activity. (A) Dependence of Pgs1 activity on the concentration of CDP-DAG, DAG, TAG, PG, dhCER, or CER (numbers denote the amounts of added lipids in µg per reaction, see Materials and Methods for details) in the reaction was measured in mitochondrial fractions isolated from the wild type cells cultivated in SMDGE medium for 24 h. Data represent mean values from at least 2 independent experiments (dots) ± SD (errorbars). Relative values normalized to the sample with the addition of 8 µg CDP-DAG are presented. (B) Regulation of Isc1 activity by PG *in vivo*. Wild type, *pgc1*Δ, *sch9*Δ and *pgc1*Δ*sch9*Δ strains of *S. cerevisiae* (see Supplementary Table for details) were cultivated in SMDGE medium without (SMDGE I-; grey columns) or with the addition of 75 µM inositol (SMDGE I+; white) for 24 h. *In vitro* activities of Isc1 were measured in isolated mitochondria. Data represent mean values from at least 6 independent experiments (dots) ± SD (errorbars). Statistically significant differences between the mutants and the wild type and/or between the cells grown without or with inositol are indicated (asterisks; ∗ – $p < 0.05$; ∗∗ – $p < 0.01$; ∗∗∗ – $p < 0.001$). WT, wild type. (C) Dependence of Pgc1 activity on the concentration of PG or DAG (numbers denote the amounts of added lipids in µg per reaction, see Materials and Methods for details) in the reaction was measured in homogenate of the *isc1*Δ cells cultivated in SMDGE medium for 24 h. Data represent mean values from at least 4 independent experiments (dots) ± SD (errorbars).

Supplementary Table. Yeast strains used in this study.

Strain	Genotype	Source
BY4741, wild type (WT)	*MAT*a *his3Δ1 leu2Δ0 met15Δ0 ura3Δ0*	Euroscarf
BY4742	*MATα his3Δ1 leu2Δ0 lys2Δ0 ura3Δ0*	Euroscarf
pgc1Δ	BY4741; *pgc1Δ::KanMX4*	Euroscarf
isc1Δ	BY4741; *isc1Δ::KanMX4*	Euroscarf
sch9Δ	BY4741; *sch9Δ::KanMX4*	Euroscarf
alpha-*pgc1Δ*	BY4742; *pgc1Δ::KanMX4*	Euroscarf
pgc1Δ::HIS3	BY4741; *pgc1Δ::HIS3*	This study
alpha-*pgc1Δ::HIS3*	BY4742; *pgc1Δ::HIS3*	This study
sch9Δ::NatMX4	BY4741; *sch9Δ::NatMX4*	This study
pgc1Δisc1Δ	BY4741; *pgc1Δ::HIS3, isc1Δ::KanMX4*	This study
isc1Δsch9Δ	BY4741; *isc1Δ::KanMX4, sch9Δ::NatMX4*	This study
pgc1Δsch9Δ	BY4741; *pgc1Δ::HIS3, sch9Δ::KanMX4*	This study
pgc1Δisc1Δsch9Δ	*MAT*a *his3Δ1 leu2Δ0 met15Δ0 ura3Δ0*; *pgc1Δ::HIS3, isc1Δ::KanMX4, sch9Δ::NatMX4*	This study
crd1Δ	*BY4741; crd1::KanMX*	Euroscarf
crd1Δ::HIS3	*BY4741; crd1::HIS3*	This study
crd1Δisc1Δ	BY4741; *crd1Δ::HIS3, isc1Δ::KanMX4*	This study
crd1Δpgc1Δ	BY4741; *crd1Δ::KanMX4, pgc1Δ::HIS3*	(31)
